# Stratification of MDD and GAD patients by resting state brain connectivity predicts cognitive bias

**DOI:** 10.1016/j.nicl.2018.04.033

**Published:** 2018-04-30

**Authors:** Janine D. Bijsterbosch, Tahereh L. Ansari, Stephen Smith, Oliver Gauld, Ondrej Zika, Sirius Boessenkool, Michael Browning, Andrea Reinecke, Sonia J. Bishop

**Affiliations:** a Centre for Functional MRI of the Brain (FMRIB), Wellcome Centre for Integrative Neuroimaging, Nuffield Department of Clinical Neurosciences, University of Oxford, John Radcliffe Hospital, Headley Way, Oxford OX3 9DU, UK; b Department of Psychology and Helen Wills Neuroscience Institute, University of California Berkeley, United States; c Department of Psychiatry, University of Oxford, UK

**Keywords:** Resting state, Connectivity, Generalized anxiety disorder, Major depressive disorder, Biomarker, Limbic, Amygdala, Attention, Stratification

## Abstract

Patients with Generalized Anxiety Disorder (GAD) and Major Depressive Disorder (MDD) show between-group comorbidity and symptom overlap, and within-group heterogeneity. Resting state functional connectivity might provide an alternate, biologically informed means by which to stratify patients with GAD or MDD. Resting state functional magnetic resonance imaging data were acquired from 23 adults with GAD, 21 adults with MDD, and 27 healthy adult control participants. We investigated whether within- or between-network connectivity indices from five resting state networks predicted scores on continuous measures of depression and anxiety. Successful predictors were used to stratify participants into two new groups. We examined whether this stratification predicted attentional bias towards threat and whether this varied between patients and controls. Depression scores were linked to elevated connectivity within a limbic network including the amygdala, hippocampus, VMPFC and subgenual ACC. Patients with GAD or MDD with high limbic connectivity showed poorer performance on an attention-to-threat task than patients with low limbic connectivity. No parallel effect was observed for control participants, resulting in an interaction of clinical status by resting state group. Our findings provide initial evidence for the external validity of stratification of MDD and GAD patients by functional connectivity markers. This stratification cuts across diagnostic boundaries and might valuably inform future intervention studies. Our findings also highlight that biomarkers of interest can have different cognitive correlates in individuals with versus without clinically significant symptomatology. This might reflect protective influences leading to resilience in some individuals but not others.

## Highlights

• Resting state fMRI markers used to re-stratify patients with GAD and MDD.
• Elevated limbic connectivity (LC) linked to high levels of depression.
• Patients with high LC showed increased attentional bias towards threat.
• LC may be a candidate marker for stratifying patients entering treatment trials.

## 1 Introduction

A quarter of the adult US population meet criteria for an anxiety or depressive disorder within a 12-month period, creating a substantial health burden for society (Kessler et al., 2005). Anxiety and depressive disorder comorbidity is high. Generalized Anxiety Disorder (GAD) and Major Depressive Disorder (MDD) show particularly extensive comorbidity and symptom overlap (Brown et al., 2001; Kessler et al., 2008). Findings also indicate significant shared genetic influences (Kendler et al., 2007), and overlapping neural substrates (Diener et al., 2012; Etkin and Schatzberg, 2011; Mochcovitch et al., 2014; van Tol et al., 2010). These findings have led to the suggestion that both disorders may share common etiological processes.

Like other psychiatric disorders, both GAD and MDD also show within diagnosis heterogeneity (Drysdale et al., 2017). The combination of between diagnosis comorbidity together with within diagnosis heterogeneity has led to increasing interest in biomarker-based stratification of patient groups. It has been argued that biomarker driven definition of patient groups might enable patient stratification to be more closely aligned to the mechanisms that are disrupted, potentially improving both outcome prediction and treatment choice (Cuthbert and Insel, 2013).

In the current study, we examined whether functional brain connectivity at rest might support an alternate stratification of patients with MDD and GAD to that determined by DSM diagnostic categorization. Until recently, most resting state studies of MDD or GAD have compared patients with a single clinical diagnosis against control participants. Considering MDD findings first, several early studies reported increased connectivity between the default mode network (DMN), subgenual anterior cingulate cortex (subgenual ACC) and thalamus in patients with MDD relative to control participants [for a meta-analysis, see Hamilton et al. (2015)]. In contrast, recent studies have found patterns of decreased as opposed to increased subgenual ACC connectivity to characterize individuals with MDD relative to healthy controls (Murrough et al., 2016; Wu et al., 2016). These inconsistencies in findings might in part reflect differences in methods adopted (e.g. seed-based versus network-based analyses and local versus global measures of connectivity), but might equally reflect MDD heterogeneity leading to variations in results across studies.

Resting state studies of patients with GAD are less numerous and have primarily focused on patterns of amygdala connectivity (Etkin et al., 2009; Li et al., 2016; Makovac et al., 2016; Roy et al., 2013). These studies have reported differences in amygdala–frontal connectivity between patients with GAD and control participants (Etkin et al., 2009; Makovac et al., 2016; Roy et al., 2013). However, whether increased or decreased connectivity is observed in the GAD group, together with the precise frontal subregion concerned, differs across studies (Hilbert et al., 2014). This might also reflect both heterogeneity within GAD patients and methodological differences between studies. Interestingly, frontal-amygdala connectivity differences have been shown to reverse in sign depending on whether GAD patients are being compared against control participants or whether correlates of continuous measures of anxiety are being examined within the GAD group (Etkin et al., 2009). This finding cannot be explained by methodological differences and as such is particularly strong evidence for within-group heterogeneity.

The studies reviewed above highlight the inconsistencies in findings within the resting state literature on MDD and GAD. An additional study directly compared resting state functional connectivity between healthy controls, patients with GAD and patients with MDD (Oathes et al., 2015). No significant resting state differences were observed between the three groups. As always it is difficult to interpret a null finding. However, the same study found scores on a continuous measure of negative affect (anxious arousal), to be linked to differential patterns of subgenual ACC activity. This raises the possibility, supported by other recent work (Drysdale et al., 2017), that identification of resting state markers linked to scores on continuous measures of affect might provide an alternate, more biologically informed means of stratifying patients with affective disorders than reliance on traditional diagnostic boundaries.

In the current study, we first used advanced MRI acquisition and analysis techniques to examine whether novel subgroups of participants with MDD and GAD could be derived by linking resting state connectivity to scores on continuous measures of affect. We next investigated whether these resting-state defined subgroups differed in cognitive function, specifically extent of attentional bias towards threat. This cognitive bias is listed as a behavior of interest under NIMH's RDoC initiative given its potential relevance to a number of psychiatric disorders. Further, attentional bias modification is one of the main targets in cognitive interventions for anxiety and depression (Amir et al., 2009; Browning et al., 2012; Heeren et al., 2015; Yang et al., 2015). However, both these intervention studies and basic investigations of attentional biases in anxiety and depression have produced mixed findings, potentially reflecting heterogeneity within patient groups (Heeren et al., 2015; Mogg et al., 2017; Mogoaşe et al., 2014; Peckham et al., 2010). Hence, identification of resting state markers that predict attentional bias towards threat might provide a valuable means of stratifying patients with GAD and MDD in the context of intervention trials. Here, the long-term goal is to advance our understanding of baseline patient characteristics that predict the success of alternate interventions.

The specific aims for our current study were as follows. First, to identify resting state markers linked to continuous measures of anxiety or depression. Second, to determine if markers so identified predict attentional bias towards threat measured at a different point in time. Here, existing work on the neural substrate of attentional bias towards threat (Bishop, 2009; Bishop et al., 2004; Bishop et al., 2007) lead us to hypothesize that increased connectivity within a limbic network including the amygdala or reduced connectivity within cingulate or executive networks or between these networks and the limbic network might be especially likely to emerge as resting state markers predictive of attentional bias towards threat. Finally, we also sought to determine whether any relationship between resting state connectivity and attentional bias towards threat is constant across both patients (with GAD or MDD) and healthy matched controls or is specific to the clinical group. Here, the former would be consistent with a trait vulnerability factor, while the latter would be consistent with protective factors being at play in the control group.

## 2 Material and methods

### 2.1 Participants

We recruited 23 participants who met diagnostic criteria for Generalized Anxiety Disorder (GAD), 21 participants who met diagnostic criteria for Major Depressive Disorder (MDD) and 27 healthy control participants. Demographic details are given in Table 1.

**Table 1:** Participant demographic details and questionnaire scores. Healthy Control participants (HC), participants with GAD and participants with MDD did not differ significantly in age, F(2,68) = 1.71, *p* = 0.19, or male or female ratio, χ^2^(2, *N* = 71) = 0.06, *p* = 0.97. Participants in the MDD and GAD groups had higher scores on the Spielberger State Trait Anxiety Inventory (STAI) trait subscale and the Beck Depression Inventory (BDI) than healthy control (HC) participants (***p < 0.0001, Bonferroni-corrected paired *t*-test.) Participants in the MDD group had higher scores on the BDI than participants with GAD (*p* < 0.0001, Bonferroni-corrected). There was no significant difference in STAI scores between the MDD and GAD groups (*p* > 0.1 Bonferroni-corrected).

Group	*N* (female)	Age		STAI		BDI	
		Mean	SD	Mean	SD	Mean	SD
HC	27 (18)	27.11	8.60	33.52	9.92	2.22	4.01
GAD	23 (16)	28.57	9.74	54.74^⁎⁎⁎^	9.27	15.43^⁎⁎⁎^	8.74
MDD	21 (14)	32.10	10.01	60.24^⁎⁎⁎^	7.35	25.59^⁎⁎⁎^	7.46

Current episode axis I DSM-IV-TR diagnoses were determined using the research version of the structured clinical interview for DSM-IV-TR (SCID) administered by staff trained and supervised by an experienced clinical psychologist. The study was approved by the Oxford Central University Research Ethics Committee (CUREC) and carried out in compliance with their guidelines. Exclusion criteria included a history of neurological disease or head injury, and psychological treatment or use of psychotropic medication within the past 3 months. We recruited an un-medicated community sample to avoid confounding influences of psychotropic medication, such as selective serotonin reuptake inhibitors, that have been associated with systematic changes in functional connectivity (McCabe et al., 2011; McCabe and Mishor, 2011; Schaefer et al., 2014). Participants who also met diagnostic criteria for OCD, PTSD, bipolar disorder, substance abuse or dependence, other anxiety disorders or eating disorders were excluded as were those showing any psychotic symptomatology. Participants who met current diagnostic criteria for both GAD and MDD were also excluded. Our hypothesis going into this study was that resting state markers would provide a potentially informative alternative subgrouping of participants with GAD and MDD to that achieved using diagnostic boundaries. We reasoned that it would be a stronger test of this hypothesis to exclude those participants currently comorbid for GAD and MDD, as their inclusion would make differentiation by DSM boundaries harder to achieve by default.

### 2.2 Procedure

Participants attended three sessions. In the first, written informed consent was obtained and the SCID conducted. In the second, participants completed standardized self-report measures of negative affect before undertaking an fMRI session comprising the resting state scan and task fMRI (not reported here). The self-report measures administered included both the Spielberger State-Trait Anxiety Inventory (STAI form Y; Spielberger, 1983) and the Beck Depression Inventory (BDI; Beck et al., 1961). Participants' scores on these scales are presented in Table 1. Additional task fMRI data were acquired in a third session. It was during this session that the attention-to-threat task was completed. A number of participants (*n* = 10, approximately 14% of our sample) either dropped out between sessions 2 and 3 or failed to fully complete session 3 and as a result did not complete the attention-to-threat task. We give the sample sizes for the resting-state analyses in Table S1 and for the resting-state against behavioral data analyses in Table S2.

### 2.3 Attention-to-threat task

We selected a task previously used to examine attentional bias towards threat under conditions of high and low perceptual load (Bishop et al., 2007). Distractor expression by perceptual load give the four conditions of interests. Participants had to determine if an ‘X’ or ‘N’ was present in a letter string superimposed on a ‘fearful’ or ‘neutral’ distractor face, as quickly and accurately as possible. In the high load perceptual condition, the letter strings comprised five non-target consonants and a single target letter; in the low perceptual load condition the letter strings comprised only target letters, i.e. 6 Ns or 6 Xs, respectively. 192 trials were presented in blocks of 4 trials, with blocks varying in the perceptual load of the letter search task. There were 24 high perceptual load blocks and 24 low perceptual load blocks; these blocks were distributed evenly across 4 runs (i.e. 6 high load and 6 low load blocks per run). In the current report, we use behavioral performance indices from this task (reaction times and error rates) as dependent measures of interest. We do not include measures of regional brain activity or connectivity during this task as additional predictors given our focus on resting state markers, which can be more feasibly translated into clinical practice.

### 2.4 Resting state fMRI acquisition and preprocessing

Fifteen minutes of resting state fMRI data (eyes open fixation) were acquired using a Siemens Verio 3 T MR system with 32-channel head coil. A whole-brain multiband EPI sequence was used (790 volumes, acceleration factor 6, TR 1140 ms, TE 40 ms, flip angle 66°, 66 slices, 2 × 2 × 2 mm voxel size), (Feinberg et al., 2010; Moeller et al., 2010; Setsompop et al., 2012). We additionally acquired fieldmaps and a T1-weighted 3D MPRAGE whole-brain structural image (TR 2040 ms, TE 4.7 ms, flip angle: 8°, voxel size 1 × 1 × 1 mm). Pre-processing was conducted using FSL (FMRIB Software Library, Version 5.00, www.fmrib.ox.ac.uk/fsl), following the Human Connectome Project standardized pre-processing pipeline (Glasser et al., 2013; Smith et al., 2013a). Preprocessing steps included motion correction, EPI unwarping, high pass filtering (cut-off full-width 2000s), functional to structural registration (using Boundary Based Registration; Greve and Fischl, 2009), and nonlinear structural to standard registration. No spatial smoothing was applied as part of the preprocessing. Confounds from participant head motion and other artefactual sources were carefully addressed by performing single-subject ICA after the preprocessing steps described above. Artefactual components were labeled using FMRIB's ICA-based X-noisefier (FIX) and all component labels were manually checked (Griffanti et al., 2014; Salimi-Khorshidi et al., 2014). Unique variance associated with artefactual ICA components and motion confounds (24 regressors: 6 motion parameters, 6 first derivatives and the squares of these 12 regressors) were removed from the data prior to conducting the connectivity analyses described below.

### 2.5 Regions of interest

**Fig. 1:**
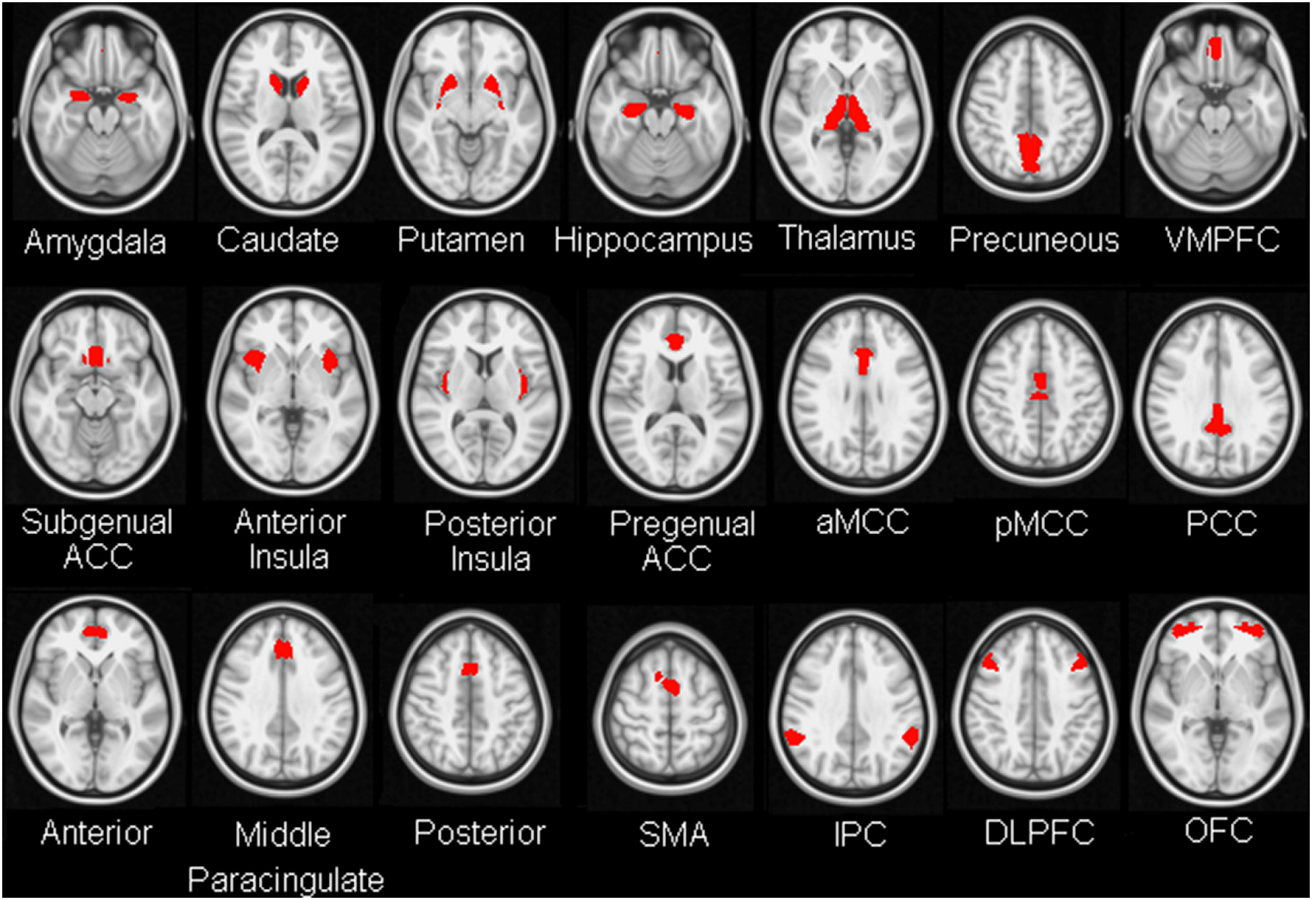
Regions of interest. The regions of interest (ROIs), adopted from our prior work on resting state correlates of trait negative affect (Bijsterbosch et al., 2014), are illustrated on a transverse image in MNI standard space. VMPC = ventromedial prefrontal cortex; ACC = anterior cingulate cortex; aMCC = anterior midcingulate cortex; pMCC = posterior midcingulate cortex; PCC = posterior cingulate cortex; SMA = supplementary motor area; IPC = intraparietal cortex; DLPFC = dorsolateral prefrontal cortex; OFC = orbitofrontal cortex.

We chose a set of 21 ROIs used in prior work by our group (Bijsterbosch et al., 2014). This set of ROIs was originally put together to span a wide range of regions implicated in emotion processing and regulation. Where possible the ROIs were defined anatomically; where this was not easily achievable, ROIs were defined functionally (Fig. 1; see also Bijsterbosch et al., 2014). Specifically, the Harvard-Oxford atlas (thresholded at 50% probability) was used to anatomically define the following regions: amygdala, caudate, putamen, hippocampus, thalamus, precuneus, ventromedial prefrontal cortex (VMPFC, medial frontal Harvard–Oxford template) and subgenual ACC (subcallosal cortex Harvard-Oxford template). The anterior insula was functionally defined using task data from a previous cohort of participants (Bijsterbosch et al., 2014), MNI x,y,z peak coordinates, left anterior insula: −38 10 −2, right anterior insula: 30 12 14. Bilateral posterior insula ROIs were obtained by subtraction of the anterior insula ROIs from the Harvard–Oxford anatomical ROIs for the insula. The Harvard–Oxford ROIs for the anterior and posterior cingulate cortex (ACC and PCC) were also further subdivided into pregenual ACC, anterior midcingulate cortex (aMCC), posterior midcingulate cortex (pMCC), and posterior cingulate cortex (PCC). The subdivisions were guided by previous work addressing this issue (Shackman et al., 2011). The boundary between pregenual ACC and the aMCC was placed at *y* = 30, the boundary between aMCC and pMCC was positioned at *y* = 4.5, and the boundary between pMCC and PCC was placed at *y* = −22. Similarly, the Harvard–Oxford ROI for the paracingulate cortex was subdivided into anterior, middle, and posterior sections. Here, as in our prior work (Bijsterbosch et al., 2014) the middle paracingulate cortex ROI was functionally defined, extending 10 mm anterior and 10 mm posterior from reported peak MNI coordinates: 0 32 36 (Kim et al., 2011). ROIs for the Supplementary Motor area (SMA, peak MNI coordinate: −6 0 58), intraparietal cortex (peak MNI coordinates, left IPC: −58 −40 38, right IPC: 54 −48 36), dorsolateral prefrontal cortex (DLPFC, peak MNI coordinates, +−40 20 34) and orbitofrontal cortex (peak MNI coordinates, left OFC: −36 52 −8, right OFC: 40 56 −4) were also defined functionally using task data from a separate cohort of participants as previously described (Bijsterbosch et al., 2014). In our prior work (Bijsterbosch et al., 2014), we used subject specific ROI redefinition to adjust ROI boundaries, prior to calculating mean time series for each ROI. In the current study, we used the principal eigen time series from each ROI, without subject-level re-definition, as this index is less affected by noise and outlier voxels (including as a result of boundary miss-specification) than the simple (unweighted) voxel average.

### 2.6 Calculation of connectivity matrices

Principal eigen time series were extracted from each ROI, and time series from bilateral regions averaged. Partial correlation with Tikhonov regularization (0.1), as implemented in FSLnets (http://fsl.fmrib.ox.ac.uk/fsl/fslwiki/FSLNets), was used to calculate Z-transformed connectivity matrices for each participant (Marrelec et al., 2006). There are two main benefits from using partial correlation to estimate connectivity matrices. First, connectivity matrices estimated using partial correlation are more sensitive to direct connections than those estimated using full correlation, the latter being influenced by indirect as well as direct connections. (Here, we note that indirect connections with nodes not included in the analysis may still exist in matrices estimated using partial correlations). Second, the use of partial correlation to estimate connectivity matrices removes global noise fluctuations that are shared between nodes (Siegel et al., 2016; Smith et al., 2013b).

**Fig. 2:**
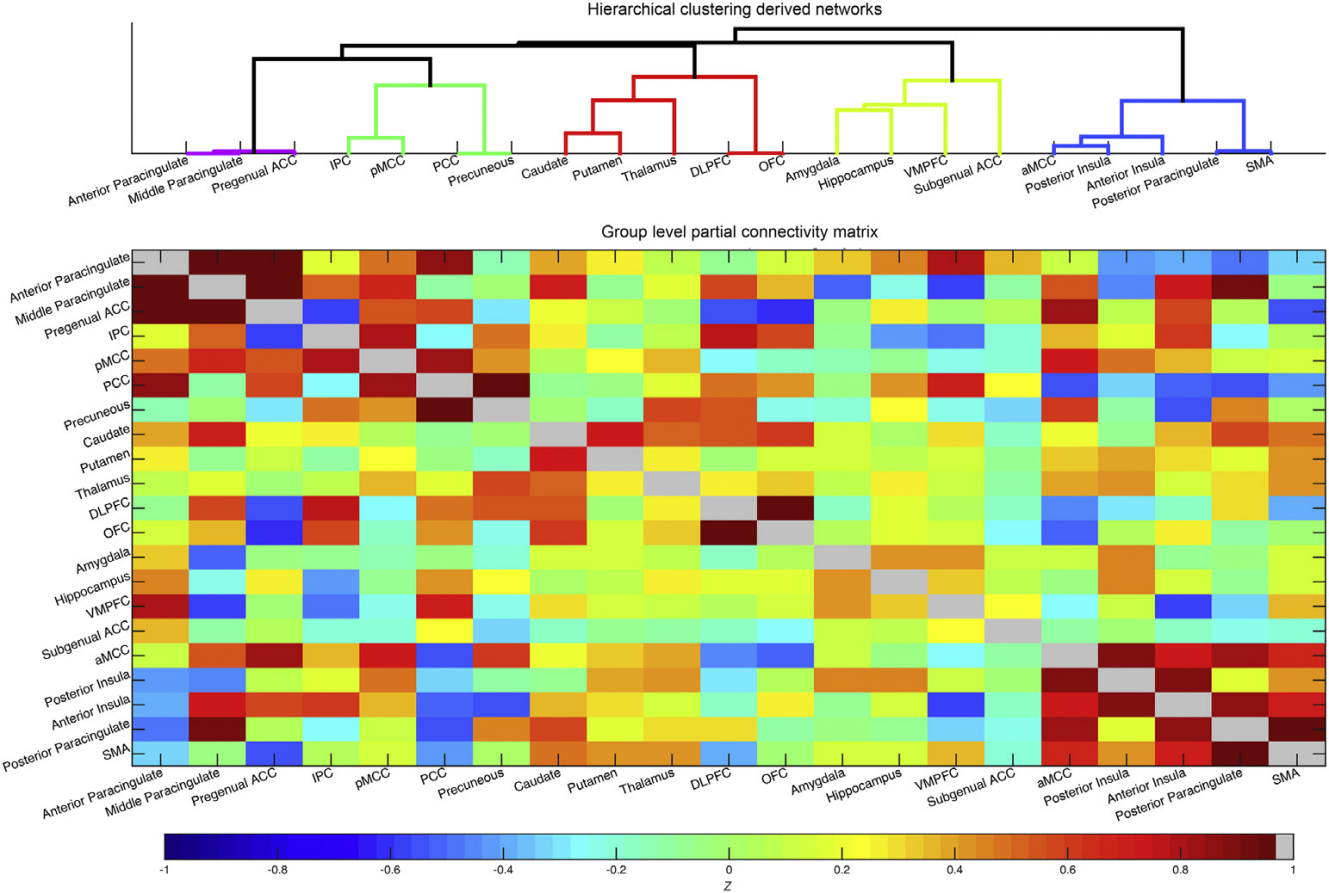
Group level functional connectivity matrix and brain networks derived by application of hierarchical nearest neighbor clustering. Partial correlation with Tikhonov regularization was performed on the principle eigen time series from all 21 ROIs, on a participant-wise basis. The group level functional connectivity matrix is shown here (bottom). Hierarchical nearest neighbor clustering applied to the group-level matrix was used to delineate networks of interest (top). A ‘paracingulate’ network comprised anterior and middle paracingulate and pregenual ACC (purple). A ‘posterior cortical-midline’ network comprised IPC, pMCC, PCC and precuneus ROIs (green). A ‘frontal-striatal network’ comprised DLPFC, OFC, thalamus, caudate and putamen (red). Amygdala, hippocampus, VMPFC and subgenual ACC formed a ‘limbic network’ (yellow). Lastly, an ‘insula-aMCC network’ (blue) comprised anterior and posterior insula, posterior paracingulate cortex, SMA and aMCC. (For interpretation of the references to color in this figure legend, the reader is referred to the web version of this article.)

The resultant functional connectivity matrices were entered (element-wise) into a one-group *t*-test to create a cross-participant functional connectivity matrix of z-transformed t-statistics. The use of t-statistics in this functional connectivity matrix accounts for variability between participants in contrast to simple averaging of subject-level matrices. Hierarchical nearest neighbor clustering was applied to this cross-participant functional connectivity matrix (Ward, 1963). Ward's minimum variance criterion minimizes the total within-cluster variance by merging clusters at each step that minimally increase the within-cluster variance. Clustering was performed using the nets_hierarchy.m Matlab code available as part of the FSLnets package (https://fsl.fmrib.ox.ac.uk/fsl/fslwiki/FSLNets/). We identified a level of the resulting tree that distinguished five networks of interest (Fig. 2). For each participant, we calculated mean within-network functional connectivity for each network by averaging the signed Z-transformed correlation between each pair of nodes within the network (resulting in 5 summary within-network measures). We also calculated the mean between-network functional connectivity for each pair of networks by averaging the signed Z-transformed correlation between each node in the first network with each node in the second network. This resulted in 10 summary between-network measures; giving 15 connectivity measures in total. We chose this level of the cluster tree as the one that best approximated known networks of interest without resulting in an unmanageable number of predictor variables (the next level up would have collapsed across the Paracingulate and Posterior Cortical - Midline networks; the next level down would have given 21 within- and between-network measures). We chose this ROI-based approach as opposed to using data-driven identification of resting state networks (e.g. ICA) to better enable comparison of regions with those discussed in the functional task literature, and to keep our methodology consistent with our prior work (Bijsterbosch et al., 2014). We note that the networks identified in this manner (Fig. 2) show considerable overlap with those identified using data-driven methodology, e.g. classical ‘salience’, ‘executive’, and ‘default’ networks (Seeley et al., 2007; Smith et al., 2009).

### 2.7 Identifying resting state predictors of anxious and depressed affect

Our first aim was to determine if any of the resting state measures, defined as detailed above, predicted participant scores on continuous measures of anxiety or depression, as indexed by the STAI trait subscale and BDI, respectively. To maximize variance on these measures we included all participants (participants with GAD, participants with MDD, and healthy control participants) in these regression analyses. Two forward stepwise regressions (*p* < 0.05 for entry and *p* > 0.10 for removal, implemented in SPSS) were conducted with the 5 within-network and 10 between-network connectivity measures entered as predictors and either STAI or BDI scores entered as dependent variables.

We note that we did not set out to identify resting state measures able to differentiate participants with GAD or MDD from healthy control participants or participants with GAD from participants with MDD. However, for completeness, two supplementary regression analyses examining whether any of the 15 resting state measures used did differentiate participants with GAD or MDD from healthy control participants or participants with GAD from participants with MDD were conducted (see Table S3).

### 2.8 Resting state driven stratification of participants

Once resting state markers that explained variance in BDI or STAI scores were identified, our second aim was to determine if re-stratifying participants using these markers would predict attentional bias towards threat. We further sought to determine (aim 3) if this relationship would differ between patients and controls. (Note we use patients from here on to refer to participants who met diagnostic criteria for GAD or MDD, it does not signify that they were under clinical care). Only one resting state measure was found to explain variance in BDI or STAI scores across participants, namely within-network limbic connectivity (this measure explaining variance in BDI scores). We hence used this limbic marker to re-stratify participants into two new groups with low or high limbic connectivity (LC), using K-means clustering (K = 2; performed using IBM SPSS Statistics software version 23). We ran this stratification across all participants to ensure that the cut-off point between low and high limbic connectivity groups was the same within patients as within controls. We then repeated the stratification within the clinical group only (excluding controls) to determine if this changed the labels that would have been given to any of the patients. It did not. We compared the four resulting groups (patients low LC; patients high LC, controls low LC; controls high LC) on three measures of head motion (mean framewise displacement, and the root mean square of the temporal derivative, DVARS, before and after ICA cleanup). There was no significant effect of resting state group, clinical status or resting state group by clinical status upon any of these three indices, *p*s > 0.2 (see Table S4 for more details).

### 2.9 Using resting-state driven stratification of participants to predict attentional bias towards threat

Finally, we examined whether participant stratification based on within network limbic connectivity (achieved using K-means clustering as described above) predicted participants' performance on the attention-to-threat task and whether this varied by participants' clinical status (patient, control). We conducted analyses of variance (ANOVAs) with either mean reaction time or error rate as the dependent variable. In both cases, two between group factors were entered: resting state group (low limbic connectivity, high limbic connectivity) and clinical status (patient or healthy control participant). For the reaction time ANOVA, within-group factors comprised perceptual load (high, low) and distractor expression (fearful, neutral). For the error rate ANOVA, we used data from the high perceptual load task condition only given the low error rates and resultant possibility of floor effects in the low perceptual load condition (see Table S5). Hence, for this analysis, there was only one within group factor: distractor expression (fearful, neutral). This three-way ANOVA revealed a significant interaction of clinical status by resting state group by distractor expression (see Results). Given this, we conducted follow-up two-way ANOVAs separately for the patient group and for controls (removing clinical status as a factor).

## 3 Results

### 3.1 Clustering of functional connectivity data

The group level functional connectivity matrix constructed from participants' ROI time-series data is shown in Fig. 2, together with the five networks identified by hierarchical clustering performed on the matrix (see Material and methods). These five networks were as follows: a limbic network comprising amygdala, hippocampus, subgenual ACC and VMPFC; a posterior cortical - midline network comprising IPC, pMCC, PCC and precuneus; a frontal-striatal network comprising regions implicated in cognitive and emotional control including DLPFC, OFC, caudate, putamen, and thalamus; an insula-cingulate network comprising regions implicated in processing stimulus saliency (Seeley et al., 2007), including posterior insula, anterior insula, aMCC, posterior paracingulate cortex, and SMA; and a cingulate-paracingulate network comprising anterior and middle paracingulate cortex and pregenual ACC. For each participant, we calculated mean within-network connectivity for each network and mean between-network connectivity for each pair of networks (see Material and methods for procedure).

### 3.2 Identifying resting state predictors of anxiety or depression scores

Linear stepwise regression analyses were conducted. The 5 within-network and 10 between-network connectivity measures were entered as independent variables and STAI trait anxiety or BDI scores as the dependent measure. With forward stepwise regression, the independent variable which most increases the fit of the regression model is added to the model at each step if it reaches significance, with further variables only being considered if they lead to a significant increase in model fit. These analyses revealed that BDI scores were positively predicted by limbic within-network connectivity, adjusted R^2^ = 0.042; F(1,42) = 4.087, standardized β = 0.236, *p* = 0.047 (Fig. S1). STAI trait anxiety scores were not significantly predicted by any of the resting state predictor variables.

### 3.3 Resting-state marker based re-stratification of participants

We next used the ‘limbic within-network connectivity’ resting state marker identified above to re-stratify participants. Specifically, we used k-means clustering to divide participants into two clusters based on limbic within-network connectivity. We performed this clustering using the whole sample (patients with GAD, patients with MDD, healthy controls). We then repeated it excluding the healthy controls. None of the patients changed their cluster membership. We hence stratified participants using the clustering results from the whole sample. This stratification both serves to re-divide the patient group according to limbic connectivity and also divides the control group at an equivalent level of limbic connectivity. This facilitates comparison of the effects of limbic connectivity (LC) based stratification across patients and controls. The mean limbic connectivity of each resulting subgroup was as follows: Patients low LC: mean = −0.06; Patients high LC mean = 1.36; Controls low LC mean = 0.04; Controls high LC mean = 1.23. As expected, given the equivalence of the k-means clustering solution when performed with versus without controls, there was no significant relationship between resting state group membership (high LC, low LC) and clinical status (patient, control): χ^2^ = 0.072, *p* = 0.811, Table S1. There was also no significant difference in resting state group membership between patients with GAD and patients with MDD, χ^2^ = 0.349, *p* = 0.763, Table S1. The resting state groups derived in this manner also showed little correspondence to groups determined by clustering participants directly on their BDI scores (χ^2^ = 0.038, *p* = 1.00). In other words, whereas the resting state measure of interest (limbic within-network connectivity) predicts depressed affect, grouping participants based on this index does not equate to grouping participants directly by depression scores.

### 3.4 Prediction of performance on attention-to-threat task

We next investigated whether participant stratification based on within-network limbic connectivity predicted performance (reaction time or error rates) on the attention-to-threat task. For the reaction time data, we conducted a four-way ANOVA with two within-subject factors: perceptual load (low versus high) and distractor expression (neutral versus fearful) and two between-subject factors: resting state group (high limbic connectivity or low limbic connectivity) and clinical status (patient or healthy controls). This ANOVA revealed that participants were faster under conditions of low versus high perceptual load (F(1,57) = 242.3, *p* < 0.0001), and when distractors were neutral as opposed to fearful (F(1,57) = 10.9, *p* = 0.002). There was also an interaction of perceptual load by distractor expression, with RT slowing on fearful, versus neutral, distractor trials primarily being observed under high perceptual load, F(1,57) = 12.2, *p* = 0.001. There were no significant interactions involving resting state group or clinical status (*p*s > 0.1). Across conditions, and across clinical status, participants with high limbic connectivity showed faster reaction times than participants with low limbic connectivity, F(1,57) = 5.8, *p* = 0.019.

Turning to the error rate analyses, the low number of errors in the low perceptual load conditions (as reported in Table S5) led to assumptions of normality being violated. Hence, we analyzed error rate data from the high perceptual load conditions only.

The analyses conducted, together with all significant (*p* < 0.05) and trend-level (*p* < 0.1) results, are summarized in Table S6. We first conducted a three-way analysis of variance with within-subject factor: distractor expression (neutral versus fearful) and between-subject factors: resting state group (high limbic connectivity or low limbic connectivity) and clinical status (patients or healthy controls). This ANOVA revealed that participants made more errors when distractors were fearful as opposed to neutral, F(1,57) = 18.5, *p* < 0.0001. There was also a significant three-way interaction of distractor expression by resting state group by clinical status, F(1,57) = 10.3 *p* = 0.002. This significant interaction indicates that the effect of grouping participants according to level of limbic within network connectivity upon attentional bias towards threat differs between patients and controls.

**Fig. 3:**
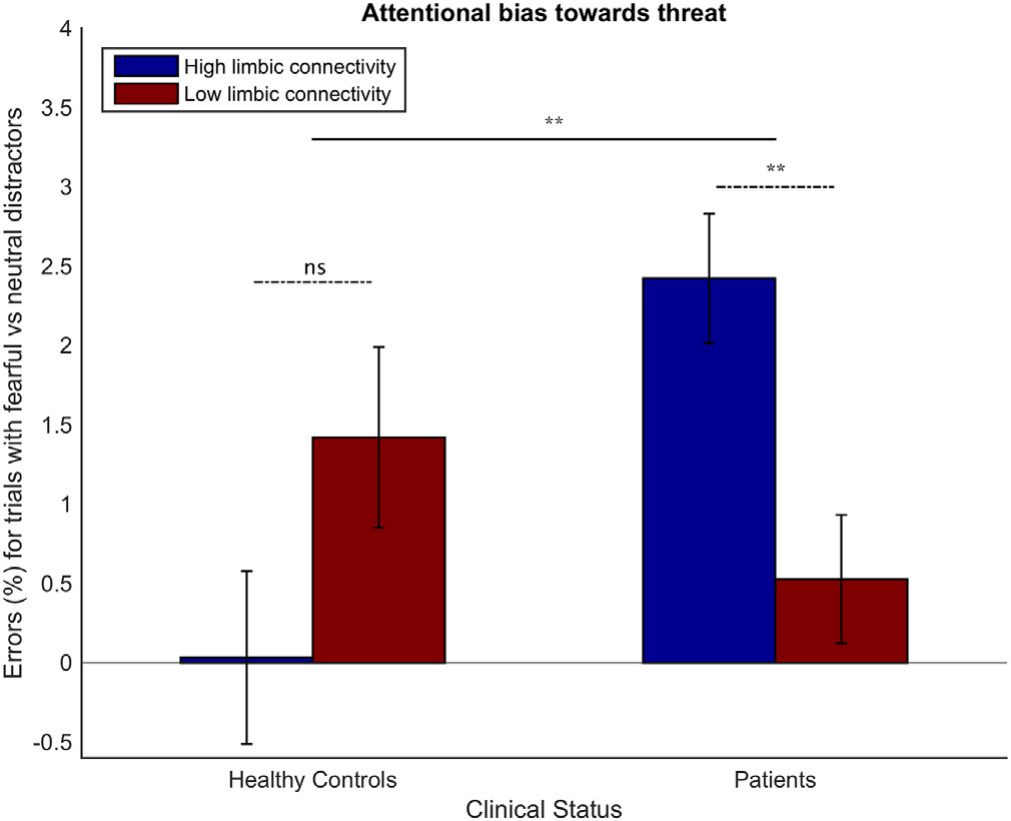
Effects of limbic connectivity at rest upon attentional bias towards threat differ between patients and healthy controls. Mean difference in error rates for trials with fearful versus neutral distractors are shown for participants grouped according to Clinical Status (Healthy Controls, Patients), and Resting State Group (high limbic connectivity, low limbic connectivity). Error bars indicate standard errors of the mean. Horizontal bars (dashed) represent interactions of Distractor Expression (fearful, neutral) by Resting State Group, shown separately for patients and controls. The solid horizontal bar represents the top-level interaction of Distractor Expression by Resting State Group by Clinical Status. (For F statistics see Results and Table S6). ** = *p* < 0.01; ns = not significant (*p* = 0.1). Note, patient is used to refer to participants diagnosed with GAD or MDD, participants are not under current psychiatric care. Data presented is for high perceptual load trials only; under low perceptual load, errors were too infrequent for analysis (see Table S5).

To break down this three-way interaction, we separated the patient and control groups and, within each of these groups, we conducted a 2-way ANOVA with error-rate as the dependent variable, resting state group as the between-participant factor, and distractor expression as the within-subject factor. In the healthy control group, there was a non-significant trend towards an effect of distractor expression, with more errors being committed on trials with fearful distractors, F(1,24) = 3.2, *p* = 0.086. There was no significant interaction of distractor expression by resting state group, F(1,24) = 2.9, *p* = 0.1 (Fig. 3). Within the patient group, there was both a highly significant main effect of distractor expression (F(1,33) = 20.9, *p* < 0.0001) and a significant interaction of distractor expression by resting state group, F(1,33) = 8.6 *p* = 0.006. Patients with high limbic connectivity showed more errors on trials with fearful, versus neutral, distractors under high perceptual load than participants with low limbic connectivity (Fig. 3).

The similar ratio of MDD and GAD participants in the high and low limbic connectivity groups (see Table S1 in addition to the non-significant Chi Square test result reported earlier) makes it highly unlikely that the interaction of distractor expression by resting state group, observed within the clinical patient group, could be merely explained by effects of DSM diagnosis (MDD vs GAD). To test this directly, we conducted a supplementary analysis on error-rate data from the clinical patient group, including diagnostic group (MDD vs GAD) as an additional between-subject factor, see Table S7. This reduced our empirical power but still revealed a significant interaction of distractor expression by resting state group (F(1,31) = 8.1, *p* = 0.008). The interaction of distractor expression by resting state group by diagnostic group was not significant, *p* > 0.5 (see Fig. S2).

## 4 Discussion

Across a combined cohort of patients with MDD, patients with GAD and healthy age and gender matched controls, BDI scores were linked to elevated connectivity within a limbic network comprising the amygdala, hippocampus, VMPFC and subgenual ACC. Stratification of participants according to this limbic connectivity index created two new subgroups that neither simply differentiated patients from controls nor patients with GAD from patients with MDD. This resting-state based stratification predicted attentional bias towards threat within patients but not within control participants. Specifically, patients with MDD or GAD with high limbic connectivity showed higher error rates on trials with fearful, versus neutral, distractors under conditions of high perceptual load than patients with MDD or GAD with low limbic connectivity (see Fig. 3). This effect remained significant when diagnostic group (MDD or GAD) was entered as a between group predictor variable. In contrast, control participants with high limbic connectivity showed, if anything, slightly less attentional bias towards threat under high load than control participants with low limbic connectivity.

These findings provide initial evidence for the external validity of resting state driven delineation of subgroups of patients with GAD and MDD. Specifically, patients with GAD or MDD characterized by high resting limbic connectivity were more likely to have threat distractors ‘break through’ and disrupt performance under task conditions which normally fully occupy participants' attention. Intriguingly, this was not the case for control group participants with high limbic resting connectivity (see Fig. 3). This suggests that there might be protective factors that offset the influence of high limbic connectivity in healthy participants, potentially not only influencing cognitive bias but also the absence versus presence of clinical symptomatology. This is likely to be a valuable avenue for future research. Similarly, further cognitively profiling those patients with GAD and MDD in the ‘low limbic’ connectivity group and determining if patients with high versus low limbic connectivity differentially benefit from treatment with SSRIs, treatment with other pharmacological agents, or cognitive interventions such as attentional bias modification will also be of importance.

To date, most resting state studies of MDD and GAD have focused on one patient group or the other. The most common methodological approach has been to use one or two seed regions of interest and to examine differences in resting state connectivity between the seed(s) and the rest of the brain for patients versus matched control participants. This has resulted in a number of publications on altered subgenual ACC connectivity in MDD and altered amygdala connectivity in GAD, though the nature and direction of reported differences has varied between studies. In the current study, we used clustering of functional connectivity at rest to identify brain networks and create a number of resting state indices or markers of interest. This approach overcomes the limitations of seed-based approaches while reducing the dimensionality of data entered into further analyses. Both the subgenual ACC and amygdala clustered together with the hippocampus and VMPFC. This limbic network was the only network where mean within-network connectivity was found to vary significantly as a function of continuous measures of depression or anxiety. Stratification of participants on this limbic connectivity index predicted attentional bias towards threat in patients with GAD and MDD but not healthy controls. This suggests that the primary value in assessing limbic connectivity might not be to obtain a trait ‘risk’ factor but rather to characterize subgroups of patients with distinct cognitive profiles. Such increased characterization of sub-groups of patients with GAD and MDD is likely in turn to be essential to the development, and assessment, of more individualized approaches to treatment.

The increased attentional bias towards threat found to characterize the high limbic connectivity patient subgroup, as indicated by increased error on trials with fearful versus neutral distractors, was observed under conditions of high perceptual load. Attentional bias towards threat has previously been reported in clinically anxious and depressed patients, and in otherwise healthy individuals with elevated trait anxiety (Bishop, 2007; Foland-Ross and Gotlib, 2012). In the latter group, increased amygdala activity, and decreased lateral prefrontal activity to threat-related, versus neutral, distractors is observed under low attentional load conditions (Bishop et al., 2007). Experimental stress manipulations have been shown to lead to a shift such that these differences in regional brain activity are predominantly observed under high load conditions, with performance also being more severely impacted by the presence of fearful distractors under these conditions (Cornwell et al., 2011). In the current study, the subgroup of MDD and GAD patients characterized by elevated limbic connectivity show a performance pattern similar to that previously reported for healthy volunteers put under experimental stress. Given the established link between limbic, especially amygdala, reactivity and attentional bias towards threat, this raises the possibility that this subgroup may show a baseline pattern of limbic reactivity that enables threat-related distractors to break through high perceptual load, in a manner otherwise primarily observed under induced stress. We note that there were insufficient errors to analyze error-rate data from the low perceptual load condition.

### 4.1 Limitations and future directions

In this work, we provide proof-of-concept evidence for stratification of GAD and MDD patients based on resting state functional connectivity indices. The subgroups identified using this data-driven stratification differed in behavioral performance on a task assessing attentional bias towards threat. This is of interest as current cognitive interventions for both GAD and MDD focus on remediating such attentional biases but have shown mixed success. A key challenge is hence to determine whether such interventions are unreliable or whether they are reliable but only effective for a subgroup of patients, and if so which individuals are most likely to benefit.

Given the modest scale of the current study, we were unable to include other patient groups which commonly show comorbidity with GAD or MDD (e.g. Obsessive Compulsive Disorder, Bipolar Disorder). Future larger scale studies would both serve to determine if the finding reported here replicate and to establish if stratification by limbic resting connectivity also predicts attentional bias to threat in these related patient groups. In addition, it would be possible to additionally explore resting state correlate of scores on measures of mania or compulsive symptomatology, as potentially validated against performance on other cognitive tasks.

In the current study, we excluded patients comorbid for GAD and MDD, as we sought to rule out the possibility that resting state markers of interest would simply follow diagnostic DSM boundaries. It would be harder to test this in the presence of diagnostic comorbidity. It is possible that we may have missed resting state markers uniquely linked to the comorbid presentation of GAD and MDD. However, we believe that this is unlikely as there was still considerable subclinical anxiety symptomatology in the MDD patient group and considerable subclinical depression symptomatology in the GAD group (see Table 1).

We note that whereas within-network limbic connectivity predicted BDI scores, we did not identify a resting state measure that predicted scores on the STAI. This might potentially reflect our moderate sample size. In prior work, we have found connectivity between prefrontal and posterior cortical - midline regions to be linked to elevated scores on a cognitive dimension of anxiety with high loadings on the STAI (Bijsterbosch et al., 2014). Hence, future larger scale studies might well identify other resting state measures of interest for further stratifying patients with GAD and MDD. In such future studies, it might be of value to consider other continuous measures of anxiety, and to separate out variance uniquely linked to anxiety versus common to scores on measures of depression.

### 4.2 Conclusions

In summary, we demonstrate how resting state functional connectivity can be used to stratify a combined group of patients with MDD and GAD. The subgroup of MDD and GAD patients characterized by elevated limbic within-network connectivity showed greater disruption by threat-related distractors of task performance under high attentional load. We hope that this data-driven stratification of patients with GAD and MDD might be of predictive value in future intervention settings. In particular, the limbic connectivity index identified here may be a strong candidate for informing both attentional bias modification trials and studies of SSRI effects on limbic reactivity to threat (Godlewska et al., 2012).

## Appendix A Supplementary data

Supplementary material Image 1

Supplementary table Image 2
