# Supplementary material for: Stratification of MDD and GAD patients by resting state brain connectivity predicts cognitive bias
Source: Neuroimage Clin. 2018 Apr 30;19:425–33. doi: 10.1016/j.nicl.2018.04.033 (PMC6051497; doi:10.1016/j.nicl.2018.04.033)
Supplement: Supplementary file 1 — Supplementary material [file mmc1.docx]

## Supplementary material

|  | **Low Limbic Connectivity** | **High Limbic Connectivity** |
| --- | --- | --- |
| **Patients** | 23 | 21 |
| **Healthy Controls** | 15 | 12 |
| **Patient sub-groups:** |  |  |
| **GAD only** | 13 | 10 |
| **MDD only** | 10 | 11 |
| **Low BDI** | 21 | 19 |
| **High BDI** | 17 | 14 |

### Table S1: Numbers of participants (n) when stratified by resting state group (low or high limbic connectivity) against clinical status (patient, healthy controls), diagnosis group (GAD, MDD), or k-means clustering on BDI scores (low BDI, high BDI).

|  | **Low Limbic Connectivity** | **High Limbic Connectivity** |
| --- | --- | --- |
| **Patients** | 18 | 17 |
| **Healthy Controls** | 14 | 12 |

### Table S2: Sample size (n) for analyses of attention-to-threat task performance as a function of resting state group (low or high limbic connectivity) and clinical status (patients, heathy controls).

| **Predictor variables** | **Subjects included** | **Dependent variable** | **Result** |
| --- | --- | --- | --- |
| 15 resting state measures | All | Clinical status  (Patient vs Healthy Control) | No resting state measures entered the model at p<0.05 |
| 15 resting state measures | Patients only | Diagnostic group  (MDD vs GAD) | No resting state measures entered the model at p<0.05 |

Table S3: Supplementary stepwise regression analyses. For completeness, we examined whether any of the within- or between- network resting state connectivity measures used in this study differentiated patients from controls or patients with MDD from patients with GAD. None of the resting state measures significantly predicted clinical status or diagnostic group. This might reflects the heterogeneity of patient groups as defined using traditional (DSM-determined) diagnostic criteria.

|  | **Head Motion Indices** | | |
| --- | --- | --- | --- |
|  | **Mean FD** | **DVARS pre ICA** | **DVARS post ICA** |
| **1. DSM based groups** |  | | |
| **GAD** | 0.18±0.06 | 377.0±42.4 | 274.8±20.0 |
| **MDD** | 0.18±0.06 | 375.7±51.4 | 271.3±22.8 |
| **Healthy Control (HC)** | 0.18±0.07 | 380.6±30.0 | 273.2±18.6 |
| One way ANOVA: | | | |
| Between-subject effect of Patient Group | F(2,68)=0.03, p=0.97 | F(2,68)=0.09, p=0.91 | F(2,68)=0.17, p=0.84 |
| **2. Groups stratified by resting state connectivity** |  | | |
| **Patient Low LC**^1^ | 0.18±0.07 | 367.1±40.1 | 273.1±18.3 |
| **Patient High LC** | 0.18±0.06 | 376.4±46.3 | 273.1±21.2 |
| **HC Low LC** | 0.19±0.07 | 373.4±37.8 | 274.3±17.7 |
| **HC High LC** | 0.18±0.06 | 383.2±43.8 | 271.9±22.8 |
| Two way ANOVA: | | | |
| Between-subject effect of resting state (Low LC, High LC) | F(1,67)=0.32, p=0.58 | F(1,67)=0.48, p=0.49 | F(1,67)=0.38, p=0.54 |
| Between-subject effect of clinical status (Patient, HC) | F(1,67)=0.001, p=0.98 | F(1,67)=0.12, p=0.73 | F(1,67)=0.002, p=0.96 |
| Interaction of resting state group x clinical status | F(1,67)=0.70, p=0.41 | F(1,67)=1.55, p=0.22 | F(1,67)=0.39, p=0.54 |

### Table S4: Head motion indices: mean scores (± standard deviation) and group comparisons. Top (1): Across several indices of head motion, there were no significant differences between patients with GAD, patients with MDD and healthy control participants. Bottom (2): There were also no significant differences in motion as a function of resting state group, clinical status or the interaction of these factors. Note: ^1^LC = Limbic Connectivity.

###

|  | Distractor Expression | |
| --- | --- | --- |
| Participant Group | Fearful | Neutral |
| **Patient Low LC**^1^ | 0.86±1.24 | 0.86±1.20 |
| **Patient High LC** | 1.98±3.95 | 2.11±4.21 |
| **Healthy Control Low LC** | 0.78±1.41 | 1.03±1.39 |
| **Healthy Control High LC** | 0.73±1.22 | 0.90±1.04 |

### Table S5. Mean percentage error rates (± standard deviation) in the low perceptual load conditions of the attention-to-threat task. ^1^LC = Limbic Connectivity

| **Analysis** | **Subjects included** | **Within-subject factor** | **Between-subject factor(s)** | **Results: significant main effects and trend-level results.** | **Results: significant interaction effects and trend-level results** |
| --- | --- | --- | --- | --- | --- |
| **1** | **All**  (n=61) | Distractor expression  (fearful vs neutral) | Resting state group:  (high LC vs low LC^1^)  Clinical status  (patient vs control) | Distractor expression: F(1,57)=18.5, p<0.0001  Clinical status:  F(1,57)=3.3, p=0.074 | Distractor expression * Resting state group * Clinical status:  F(1,57)=10.3, p=0.002 |
| **2** | **Patients only**  (n=35) | Distractor expression  (fearful vs neutral) | Resting state group  (high LC vs low LC) | Distractor expression: F(1,33)=20.9, p<0.0001 | Distractor expression * Resting state group:  F(1,33)=8.6, p=0.006  Distractor expression, post-hoc t-tests:  High LC sub-group: *t*=5.3, p<0.0001  Low LC sub-group: *t*=1.2, p=0.27 |
| **3** | **Healthy controls only**  (n=26) | Distractor expression  (fearful vs neutral) | Resting state group  (high LC vs low LC) | Distractor expression:  F(1,24)=3.2, p=0.086 | Distractor expression * Resting state group  F(1,24)=2.9, p=0.1  ^2^Distractor expression, post-hoc t-tests:  High LC sub-group: *t*=0.06, p=0.95  Low LC sub-group: *t*=2.4, p=0.03 |

### Table S6: Overview of findings from analyses of variance (ANOVAs) with error rates on the attention-to-threat task as the dependent measure of interest. The initial ANOVA (1) gave a significant three-way interaction of distractor expression * resting state group* clinical status. Hence we broke this down by conducting two separate follow-up analyses on data from (2) Patients only and (3) Healthy controls only. Note. Only data from the high perceptual load conditions were analyzed given the small number of errors in the low perceptual load conditions (see Table S5). ^1^LC = Limbic Connectivity. ^2^ Note, the interaction term was not significant. We add these post-hoc tests for comparison against those reported in (2).

| **Analysis** | **Subjects included** | **Within-subject factor** | **Between-subject factor(s)** | **Results: significant main effects and trend-level results.** | **Results: significant interaction effects and trend-level results** |
| --- | --- | --- | --- | --- | --- |
| **S1** | **Patients only**  (n=35) | Distractor expression  (fearful vs neutral) | Resting state group  (high LC vs low LC^1^)  Diagnostic group  (MDD vs GAD) | Distractor expression: F(1,31)=19.0, p<0.0001 | Distractor expression * Resting state group  F(1,31)=8.1, p=0.008  Distractor expression * Diagnostic Group:  F(1,31)=3.76, p=0.062 |

### Table S7: For the patient group, we conducted a supplementary analysis of variance upon error rates on the attention-to-threat task under conditions of high perceptual load including diagnostic group (GAD, MDD) as a variable of interest. We retained resting state group (low limbic connectivity, high limbic connectivity) as a between-subject factor and distractor expression (neutral, fearful) as a within-subject factor. We included DSM diagnostic group (GAD, MDD) as an additional between-subject factor to examine whether the effect of resting state group upon error rates for fearful versus neutral distractor trials would remain robust to the inclusion of this variable. This was indeed the case; a significant two-way interaction of distractor expression x resting state group was observed (p=.008); patients with high limbic connectivity showed higher rates of errors on trials with fearful versus neutral distractors, as in the analyses reported in the main manuscript. This two-way interaction was not moderated by a three way interaction with diagnostic group (distractor expression x resting state group x diagnostic group, F(1,31)= 0.3; p=0.57). Here, we note that the sample sizes for this supplementary analysis were small so this three-way interaction was under-powered (GAD High LC: n=10 ; GAD Low LC n =10 ; MDD High LC: n=7; MDD Low LC :n= 8). Note. ^1^LC = Limbic Connectivity.

###

### Figure S1: Scatterplot of Beck Depression Inventory (BDI) scores against Limbic within-network functional connectivity


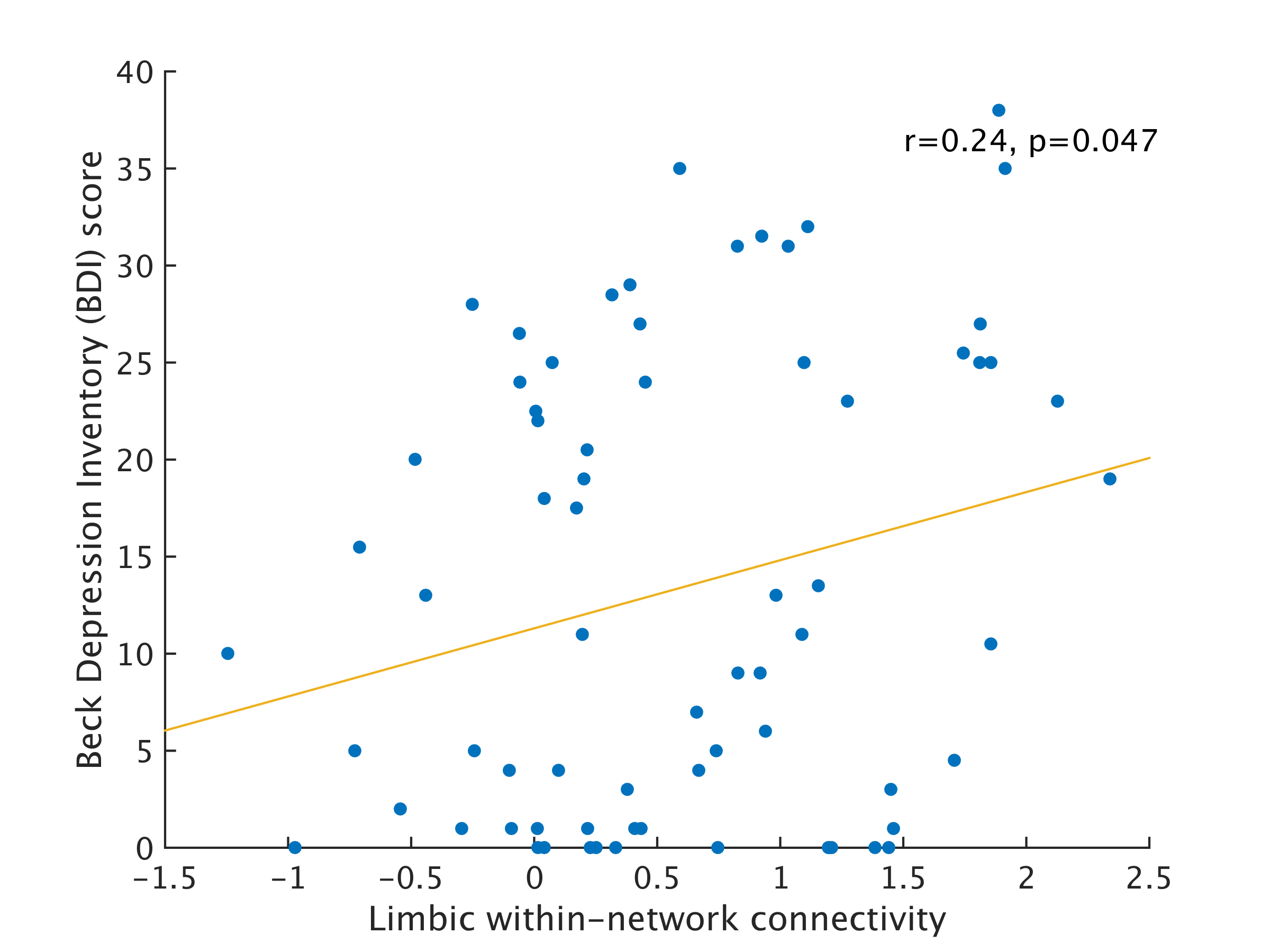


**Figure S2.**

**
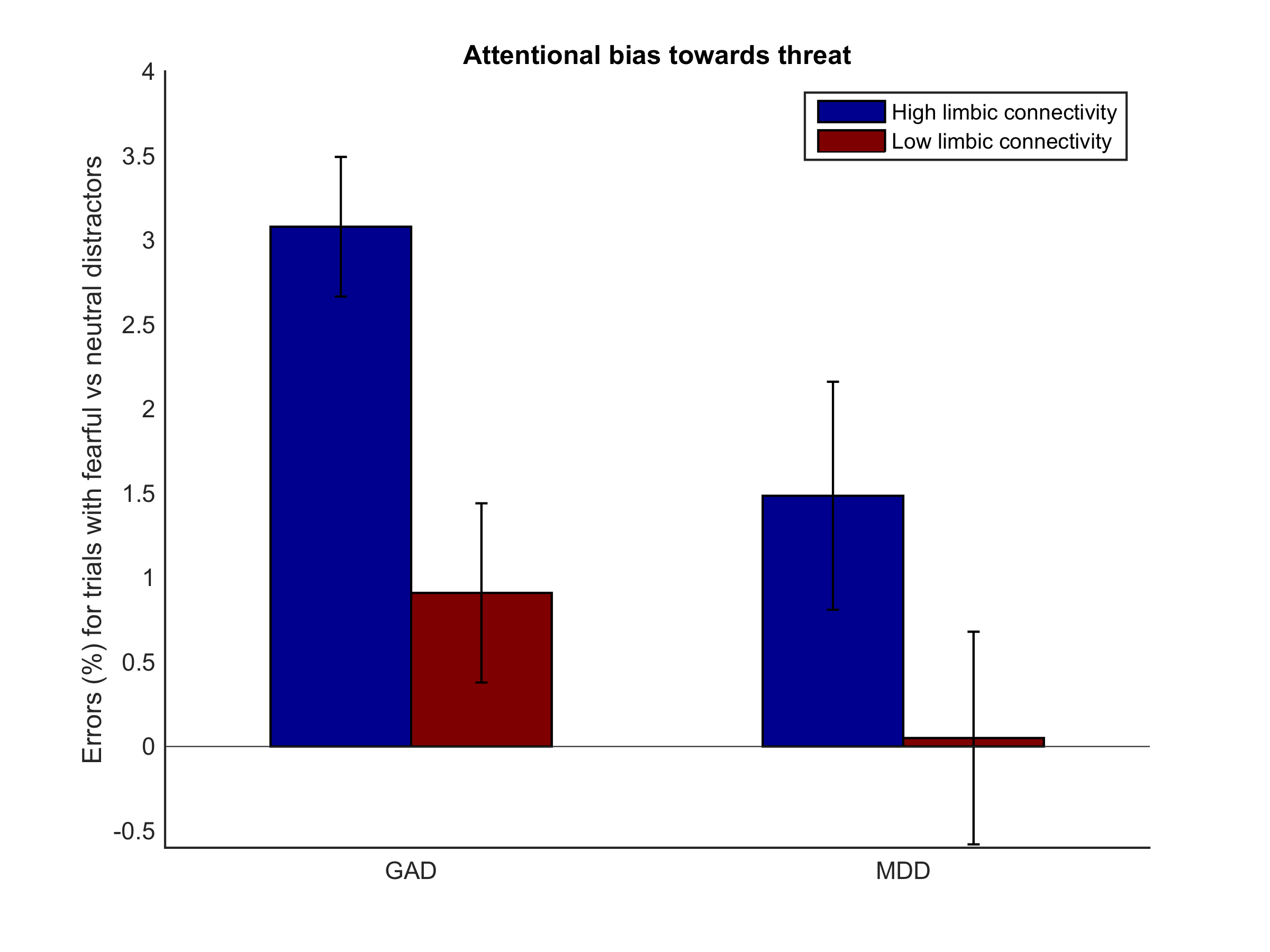
**

**Effects of limbic within-network functional connectivity, at rest, upon attentional bias towards threat within patients with GAD and MDD.** This figure illustrates the results of our supplementary error-rate analysis including diagnostic group as a between-subject factor (see Table S7 for further details). Mean difference in error rates for trials with fearful versus neutral distractors are shown for participants grouped by diagnostic group (GAD, MDD) and resting state group (high limbic connectivity, low limbic connectivity). Error bars indicate standard errors of the mean. Across patients, there was a significant interaction of distractor expression (fearful, neutral) by resting state group (high limbic connectivity, low limbic connectivity), F(1,31)=8.1, p=0.008. This was not qualified by a significant three-way interaction with diagnostic group, (F(1,31)= 0.3; p=0.57, p>.5. There was a non-significant trend towards an interaction of diagnostic group (GAD, MDD) by distractor expression, F(1,31)=3.76, p=0.062. Note, data presented is for high perceptual load trials only; under low perceptual load, errors were too infrequent for analysis.
